# Treatment of Recurrent Primary Cutaneous Mucinous Carcinoma of the Eyelid with Modified Wide Local Excision

**DOI:** 10.1155/2020/6668640

**Published:** 2020-12-18

**Authors:** Stephanie M. Tillit, Siva S. R. Iyer, Eric J. Grieser, John T. LiVecchi

**Affiliations:** ^1^College of Medicine, University of Florida, Gainesville, FL, USA; ^2^Department of Ophthalmology, University of Florida, Gainesville, FL, USA

## Abstract

Primary cutaneous mucinous carcinoma (PCMC) is a rare, low-grade malignant neoplasm of the sweat gland, whose history has been controversial regarding eccrine versus apocrine origin. This case report describes a 53-year-old male who presented to the University of Florida, Gainesville, ophthalmology clinic and was referred to the oculoplastics service with a painless, subcentimeter mass on the lateral right upper eyelid including the canthus, consistent with recurrent primary cutaneous mucinous carcinoma of the eyelid. Four years prior, the patient had undergone excisional biopsy of the lesion in a clinic, which revealed residual tumor, but the patient deferred further surgery at the time. The patient underwent surgical excision of the mass with reconstruction without operative complications and with negative surgical margins. PCMC is difficult to diagnose clinically due to its rarity and requires a histopathological examination for confirmation of the diagnosis. This report presents the first case in the literature of primary cutaneous mucinous carcinoma in a patient with human immunodeficiency virus (HIV). With this case report, we aim to raise awareness of primary cutaneous mucinous carcinoma as a potential part of the differential diagnosis for malignant eyelid lesions, including those present in patients with HIV.

## 1. Introduction

Carcinomas of the sweat gland are known to be rare malignancies with locally aggressive features and high rates of recurrence [1]. These have been traditionally subdivided into the groups of eccrine, apocrine, mixed origin (eccrine and apocrine) and other unclassifiable sweat gland tumors [1]. There is a lack of consistent immunohistochemical markers identified in these, as well as histological similarities to other malignancies, which can make diagnosis difficult. For example, primary cutaneous mucinous carcinomas (PCMCs) may resemble mucinous carcinomas found elsewhere in the body (e.g., breast, lung, gastrointestinal-type tumors) [2]. For this reason, one must rule out the possibility of metastatic lesions and correlate clinically. Herein, we report the case of a 53-year-old male with recurrent primary cutaneous mucinous carcinoma of the right upper eyelid, including diagnosis, management, and follow-up. This is the first case of PCMC in a patient with human immunodeficiency virus (HIV) reported in the literature.

## 2. Case

A 53-year-old male presented to the ophthalmology clinic for a right upper eyelid mass that had been subjectively growing over the last year. Past medical history was significant for hypertension and human immunodeficiency virus (HIV). Past ocular history was significant for primary cutaneous mucinous carcinoma of the right upper eyelid and bilateral senile nuclear sclerosis.

Four years prior, the patient had presented to the University of Florida, Gainesville, ophthalmology clinic for a painless right upper lid mass, which initially was diagnosed as a chalazion. The mass failed to improve with conservative therapy, and excision of the lesion was performed in a clinic, with pathology showing histologic features compatible with mucinous carcinoma. The tumor cells were immunoreactive for Cytokeratin 7 and Gross Cystic Disease Fluid Protein 15 (GCDFP-15) and negative for Cytokeratin 5/6 and p63. The mucin infiltrated and dissected through the fibrotic stroma and appeared to extend to the deep resection margin. Metastatic workup, including CT of the chest, abdomen, and pelvis, was negative for other disease. Though the patient was counseled on the high likelihood of recurrence, he declined any further surgery at the time. The patient followed up for one year after excisional biopsy but was subsequently lost to follow-up.

**Figure 1:**
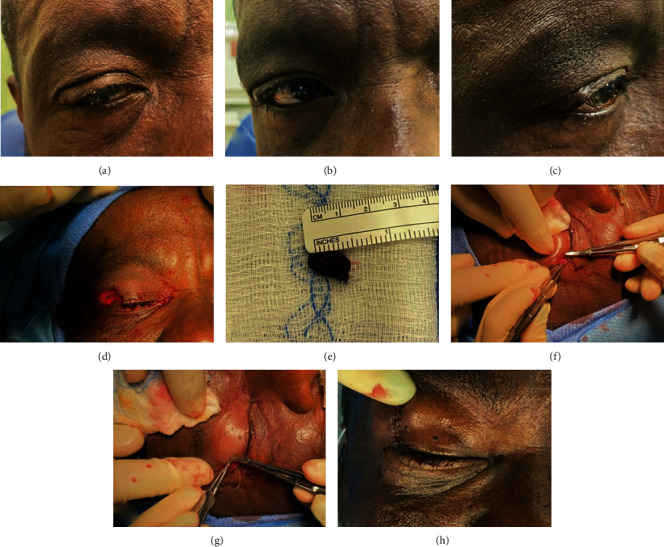
Preoperative, perioperative, and postoperative images: (a–c) preoperative photographs of patient's right upper eyelid lesion; (d) site of surgical excision postremoval of visible tumor (e) and margins; (f, g) reconstruction of excisional site including performing undermining of the two sides of the excisional site creating a sliding flap; (h) repaired site of surgical excision immediately postoperation.

Three years later, he presented to the oculoplastics clinic with a 4 mm × 5 mm mass on the lateral right upper eyelid including the canthus, which had subjectively gradually grown over the last year per the patient, without any soreness or drainage of the lesion (Figures 1(a)–1(c)). Slit lamp exam showed a right upper lid lesion, bilateral blepharitis, bilateral pigmentation and pinguecula of the conjunctiva, and bilateral senile nuclear sclerosis. A diagnostic incisional biopsy of the lesion in clinic revealed recurrent mucinous carcinoma.

The patient subsequently underwent surgical excision of the mucinous carcinoma of the right upper lid with reconstruction. There is currently no standard of care for the surgical treatment of PCMC, and interventions range from standard excision to wide local excision and Mohs surgery. [3] Herein, we describe our modified wide local excision technique to avoid significant morbidity in this patient with lid margin and lateral canthal involvement.

The right upper eyelid was inspected, and a pentagonal wedge of tissue including the carcinoma lesion was excised using an 11 blade and Westcott scissors. The lesion measured approximately 15 mm × 10 mm and was labelled as the “main” specimen with the medial aspect marked with a single suture. Margin procurement was completed with the aim of achieving satisfactory disease-free margins while retaining adequate postsurgical function. Initial full-length margins 2.5 mm wide were excised medially, temporally, superiorly, and deep. These were followed by a second set of additional excisions 5 mm wide medially, temporally, superiorly, and deep, making the total margin taken at least 7.5 mm. The outermost, or second excised, margins were sent to pathology labelled with their respective location.

The tarsus was reapproximated using a double-armed 6-0 Vicryl suture in a horizontal mattress suture. A single interrupted 6-0 Vicryl suture was used to reapproximate the anterior border of the tarsus. A lateral canthotomy was performed using Stevens scissors to make the lateral lid more mobile for closure. The superior tarsus and remaining skin defects were closed using 6-0 plain gut. Adequate hemostasis was obtained, and there was minimal loss of blood with no immediate postoperative complications.

**Figure 2:**
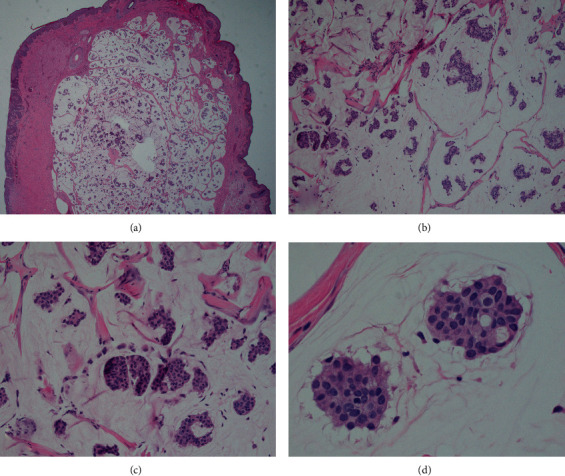
Histologic examination of the surgical specimen. Histology slide analysis was consistent with cutaneous mucinous carcinoma, including large pools of basophilic mucin with islands of relatively bland epithelioid cells with abundant cytoplasm. The cells were arranged in nests and duct/glandular-like patterns and could be seen floating within the mucin. Mitotic figures were rare. (a) 2x, (b) 10x, (c) 40x, and (d) 60x.

A histological description of the specimen is provided in Figure 2. Furthermore, tumor cells were immunoreactive for Cytokeratin 7 and GCDFP-15 and negative for Cytokeratin 5/6. Pathologic evaluation revealed negative margins around the site of excision medially, temporally, superiorly, and deep. At his one-week follow-up appointment, the patient had some trace swelling of the right upper eyelid with mild inflammation-induced ptosis and scabbing but reported neither changes in visual acuity nor discomfort. Six months after surgical excision with negative margins, the patient remained asymptomatic with no evidence of local recurrence.

## 3. Discussion

Sweat gland tumors are characterized by their locally aggressive behavior and high rate of recurrence [1]. The subset of eccrine carcinomas accounts for less than 0.01% of diagnosed cutaneous malignancies, with mucinous carcinoma being only one of the many subtypes of malignant entities [1]. Primary cutaneous mucinous carcinoma is a rare low-grade malignant neoplasm that was originally believed to be only of eccrine origin. It is now known that these tumors demonstrate apocrine-type differentiation as well, which indicates that the traditionally used term “mucinous eccrine carcinoma” is actually a misnomer [3].

PCMCs predominantly affect males and most often occur in the seventh decade of life but can be seen earlier [3]. Clinically, these are slow growing and may arise on a variety of locations, including the face (with a predominance of the eyelids), axilla, scalp, and trunk [3]. A 2014 systematic review and meta-analysis by Kamalpour et al. noted approximately 215 cases of PCMC reported in the literature, with 79 (36.7%) of these involving the eyelid/canthus/brow area [3]. Per Rismiller et al. in their 2019 population-based data analysis, there were 411 cases of PCMC reported in the National Cancer Institute's Surveillance, Epidemiology, and End Results (SEER) database from 1972 to 2013, with 159 (38.7%) of these involving the eyelid as the primary site [4].

The histopathological features of primary cutaneous mucinous carcinoma include the presence of nests and strands of epithelial cells imbedded in abundant pools of mucin, separated from each other by thin fibrous septa. Positivity for markers such as GCDFP-15 and human milk fat globulin supports an apocrine lineage, which debunked previous schools of thought [5]. Due to the rarity of these lesions, they are difficult to diagnose and are clinically confused with other lesions such as basal cell carcinoma, epidermoid cysts, Kaposi sarcoma, neuromas, lacrimal sac tumors, squamous cell carcinoma, granulomatous tumors [6], sebaceous gland carcinoma, Merkel cell carcinoma, and metastatic adenocarcinoma. In order to differentiate PCMC from metastasis of mucinous carcinoma, a thorough clinical and imaging assessment must be completed; however, some features that may suggest a primary cutaneous origin include location on head/neck region and presence of an in situ component. A proposed precursor to PCMC is endocrine mucin-producing sweat gland carcinoma (EMPSGC), which is differentiated from PCMC by demonstrating little mucin deposition extracellularly [5].

With regard to the eyelid specifically, a large retrospective study completed by Kersten et al. assessed the accuracy of clinical diagnoses of cutaneous eyelid lesions and concluded that malignant eyelid lesions can masquerade as a number of different clinically benign conditions. Such conditions include melanocytic nevus, hidrocystoma, chalazion, molluscum contagiosum, amyloidosis, capillary hemangioma, cavernous hemangioma, syringoma, telangiectasia, xanthelasma, dermal fibrosis, trichoepithelioma, sebaceous hyperplasia, sebaceous adenoma, sarcoid, pyogenic granuloma, papilloma, seborrheic keratoses, verrucae, fibroepithelial polyp, and the aforementioned epidermoid cyst. This study established that histologic confirmation is essential, because it is not possible to obtain complete accuracy in diagnosing eyelid lesions on clinical grounds alone. Repeat biopsy was recommended if the initial histopathologic evaluation did not agree with the malignant clinical diagnosis [7].

The majority of patients seem to have only local disease [4], and morbidity of PCMC is associated with incomplete resection [8, 9]. This statement is further supported by this case, in which initially, the tumor extended to the deep resection margin of the original excisional biopsy. Unfortunately, because the patient was opposed to further surgery at the time, this was most likely the nidus for the recurrence of the lesion. Tumor size over 1.5 cm is associated with increased risk of regional disease, but eyelid tumors are more likely to present with distant disease compared to other locations on the head and neck [4]. Even though the age-adjusted incidence of PCMC is 2.4-fold higher in blacks than Caucasians, PCMC-specific mortality is independent of many factors such as sex, age, race, primary site, histologic tumor grade, tumor size, tumor stage, or treatment [4].

As previously acknowledged, there is currently no standard of care for the surgical treatment of cutaneous mucinous carcinoma. Specific descriptions of local excisions, especially regarding margins in particular, have not been well-described in the literature. The SEER database analysis described wide excision versus nonwide excision as ≥1.0 cm and <1.0 cm, respectively [4]. Some sources have recommended wider margins of at least 10 mm for procedures without intraoperative frozen section control, but no studies have confirmed this as a standard margin that prevents recurrence. Location of the lesion and impact on function must also be taken into account, as taking such large margins in the periorbital area can add significant morbidity and be cosmetically disfiguring. Complications of upper eyelid repair may include and are not limited to notching, trichiasis, conjunctivalization, entropion, retraction, ptosis, and ectropion [10].

Eyelid tumors of this kind are less likely to undergo wide local excision and are more frequently treated with Mohs micrographic surgery [4], most likely due to the aforementioned potential complications. Intervention with Mohs surgery or excision with frozen section control usually reports better outcomes with only 7% recurrence rate [11]. There is a 30-40% recurrence rate with surgical removal without intraoperative evaluations of surgical margins [11]. However, the type of surgery used does not correlate with survival [4]. Long-term follow-up is essential due to the recurrent nature of PCMC, with the SEER database analysis reporting an average follow-up length of 88.2 months [4].

## 4. Conclusion

In the age of combination antiretroviral therapy, patients with HIV have a decreased incidence of AIDS-defining cancers; however, the same cannot be said about melanoma and nonmelanoma skin cancers [12]. Non-AIDS-defining cancers are usually treated according to the standard of care [13]. In this patient with a non-AIDS-defining cancer, the first reported in the literature with PCMC, the lack of a standard of care regarding management of this lesion is evident. Due to the rarity of PCMC, there have been no randomized control trial studies to directly compare the effects of wide local excision with or without frozen section control versus Mohs surgery on recurrence rates, nor to determine a definite desired clear margin length. In this case, a modified wide local excision, in the context of involvement of the lid margin and lateral canthus, was performed with adequate safety margins of at least 5 mm resulting in the preservation of function and avoidance of postsurgical morbidity. In this patient with a relatively small tumor, who had previously deferred Mohs surgery, modified wide local excision was a viable alternative which achieved clear margins and prevented recurrence up to six months after excision. Due to the favorable margins obtained in this case, there is hopeful resolution of this recurrent primary cutaneous mucinous carcinoma of the eyelid; however, long-term follow-up will be necessary.

## Data Availability

The literature review data used to support the findings of this study are included within the article.

## References

[1] Kaseb H., Babiker H. M. (2020). *Cancer, an overview of eccrine carcinoma*.

[2] Gowda K. K., Agarwal P., Bal A. (2014). Mucinous eccrine carcinoma of the eyelid: re-emphasizing the need for awareness of rare lesions. *Dermatology Reports*.

[3] Kamalpour L., Brindise R. T., Nodzenski M., Bach D. Q., Veledar E., Alam M. (2014). Primary cutaneous mucinous carcinoma: a systematic review and meta-analysis of outcomes after surgery. *JAMA Dermatology*.

[4] Rismiller K. P., Crowe D. R., Knackstedt T. J. (2020). Prognostic factors, treatment, and survival in primary cutaneous mucinous carcinoma: a SEER database analysis. *Dermatologic Surgery*.

[5] Cardoso J. C., Calonje E. (2015). Malignant sweat gland tumours: an update. *Histopathology*.

[6] Marrazzo G., Thorpe R. B., Black W. H. (2017). Primary mucinous carcinoma of the eyelid treated with Mohs micrographic surgery. *Cutis*.

[7] Kersten R. C., Ewing-Chow D., Kulwin D. R., Gallon M. (1997). Accuracy of clinical diagnosis of cutaneous eyelid lesions. *Ophthalmology*.

[8] Wright J. D., Font R. L. (1979). Mucinous sweat gland adenocarcinoma of eyelid: a clinicopathologic study of 21 cases with histochemical and electron microscopic observations. *Cancer*.

[9] Snow S. N., Reizner G. T. (1992). Mucinous eccrine carcinoma of the eyelid. *Cancer*.

[10] O'Donnell B. A., Mannor G. E. (2009). Oculoplastic surgery for upper eyelid reconstruction after cutaneous carcinoma. *International Ophthalmology Clinics*.

[11] Papalas J. A., Proia A. D. (2010). Primary mucinous carcinoma of the eyelid: a clinicopathologic and immunohistochemical study of 4 cases and an update on recurrence rates. *Archives of Ophthalmology*.

[12] Latini A., Alei L., Magri F., Eibenschutz L., Cota C. (2020). Nonmelanoma skin cancer and melanoma in HIV-1-infected patients. *AIDS*.

[13] Yarchoan R., Uldrick T. S. (2018). HIV-associated cancers and related diseases. *The New England Journal of Medicine*.

